# Genetic strategies to understand physiological pathways regulating body weight

**DOI:** 10.1007/s00335-014-9541-z

**Published:** 2014-08-26

**Authors:** Sadaf Farooqi

**Affiliations:** Addenbrooke’s Hospital, Wellcome Trust-MRC Institute of Metabolic Science, University of Cambridge Metabolic Research Laboratories, Cambridge, UK

## Abstract

Body weight is a highly heritable trait across species. In humans, genetic variation plays a major role in determining the inter-individual differences in susceptibility or resistance to environmental factors which influence energy intake and expenditure. In this review, I discuss how genetic studies have contributed to our understanding of the central pathways that govern energy homeostasis. The study of individuals harboring highly penetrant genetic variants that disrupt the leptin–melanocortin pathway has informed our understanding of the physiological pathways involved in mammalian energy homeostasis.

## Introduction

In humans, there is a strong evidence that within a population the variance in body weight and in body mass index (BMI; weight in kg/height in meters squared) is strongly influenced by genetic factors (Maes et al. 1997), with family, twin and adoption studies yielding heritability estimates that range between 40 and 70 % (Stunkard et al. 1986, 1990; Sorensen et al. 1989). In experimental overfeeding studies, Bouchard and colleagues showed that weight gain induced by overfeeding identical male twin pairs by 10 % of their energy requirements differed across sets of twins but was similar between members of a twin pair (Bouchard et al. 1990). Similarly, the response to negative energy balance was also heritable when the same individuals were calorically restricted (Bouchard et al. 1996). Given the high heritability of body weight, genetic approaches can be a useful tool with which to dissect the mechanisms involved in energy homeostasis. A number of contrasting experimental approaches have been used to identify human obesity-associated genes.

## Finding common genetic variants that are associated with increased risk of obesity

Genome-wide association studies (GWASs) are based on the premise that the heritability of common diseases is made up of a large number of common variants [minor allele frequency (MAF) of more than 5 %]. To this end, the use of high throughput arrays has facilitated the genotyping of thousands of common variants covering more than 75 % of the genome in large population-based cohorts on whom body mass index data is available. The first GWAS-derived loci detected were intronic variants in the *FTO* (fat mass and obesity-associated) gene (Frayling et al. 2007; Dina et al. 2007) and variants approximately 200 kb downstream of MC4R (Loos et al. 2008). To date, more than 50 genetic loci relevant for body weight regulation have been identified by GWAS approaches (Loos 2012). While GWAS-associated signals are often identified by the name of the nearest gene, there is little evidence to suggest that variation in these specific genes explains the association signal. Furthermore, many of the signals identified to date map to non-coding regions of the genome so their functional significance needs to be determined. The strongest association with BMI in individuals of Caucasian origin has consistently been found with SNPs in the first intron of FTO. Similar observations have been made in other ethnic groups (Zhang et al. 2008; Li et al. 2008). Homozygotes for the risk allele of the most common SNP in FTO are 3–4 kg heavier than those without the risk allele and have a 1.67-fold increased risk of obesity (Frayling 2007). Several human studies have demonstrated an association between SNPs in FTO and energy intake rather than energy expenditure (Wardle et al. 2008). FTO is expressed in the hypothalamus, and its expression has been suggested to be nutritionally regulated by some (although not all) groups (Gerken et al. 2007; Stratigopoulos et al. 2008). *Fto*-null mice are small with increased energy expenditure (Fischer et al. 2009), although the effect is negligible when using the linear regression method to correct for differences in lean mass (McMurray et al. 2013). In contrast, mice overexpressing *Fto* are obese (Church et al. 2010). Genes within the region of interest are evaluated as potential candidates for causing the association such as RPGRIP1L (Stratigopoulos et al. 2011, 2014), but the association may also arise due to long-range genetic interactions as evidenced recently by work which suggests that the nearby gene IRX3 may be important (Smemo et al. 2014).

Cumulatively, the common variants identified in GWASs are characterized by modest effect sizes (per-allele odds ratios between 1.2 and 1.5), and the proportion of variability explained by GWAS-identified loci to date remains relatively modest (<5 %). Studies in childhood onset obesity and in severely obese children and adults have shown that there is some overlap between the common variants that contribute to early-onset and adult-onset weight gain (Wheeler et al. 2013), but also that both these approaches can identify novel variants that are not found in adult populations (Bradfield et al. 2012). Differences in genetic architecture as well as in environmental confounders may explain some of the discordance between different GWAS studies. While meta-analyses of even larger datasets are underway, there is a growing consensus in the study of common complex diseases that additional common variants are unlikely to explain the missing heritability underlying traits such as BMI or obesity. There is the possibility that the heritability of obesity-related phenotypes may have been overestimated, as the effects of the shared environment and, in the case of twins, the shared in utero environment, are difficult to separate from inherited influences.

## Candidate gene studies

Candidate gene studies based on the molecules known to cause severe obesity in experimental animals have shown that these genes also contribute to childhood onset human obesity. The foundation for this work was laid by elegant physiological studies showing that the regulation of body weight is a homeostatic process (Friedman 2000), which has been demonstrated by lesioning studies to be regulated at the level of the hypothalamus. Parabiosis experiments in inbred strains of mice with severe obesity, such as *ob/ob* and *db/db*, suggested the existence of a circulating factor that regulates weight which was missing in *ob/ob* mice and to which *db/db* were resistant (Coleman and Hummel 1969). The identification of this hormone, leptin, through positional cloning of the *ob* gene, and the finding that this was mutated in severely obese *ob/ob* mice (Zhang et al. 1994) paved the way for the molecular and physiological circuits controlling energy homeostasis to be dissected. Leptin is a 16-kDa hormone whose circulating levels correlate closely with fat mass. Many of the physiological effects of leptin are mediated through the central nervous system, particularly the hypothalamus, which is the site of the highest mRNA expression of the long signaling isoform of the leptin receptor. Leptin stimulates pro-opiomelanocortin (POMC) expressing primary neurons in the arcuate nucleus of the hypothalamus. POMC is post-translationally processed to yield the melanocortin peptides, which are agonists at melanocortin 3 and 4 receptors. In addition, leptin inhibits neurons expressing the melanocortin antagonist Agouti-related protein and neuropeptide Y (NPY); NPY can suppress the expression of POMC. These primary leptin-responsive neurons project to second-order neurons expressing the melanocortin 4 receptor (MC4R). Targeted genetic disruption of MC4R in mice leads to increased food intake and increased lean mass and linear growth (Huszar et al. 1997). These hypothalamic pathways interact with other brain centers to coordinate energy intake and energy expenditure (Cummings and Schwartz 2003).

## Insights from genetics—leptin signaling

**Fig. 1:**
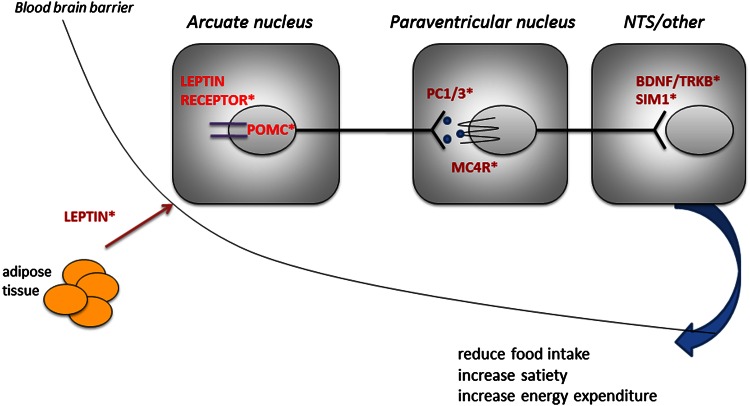
Schematic of the hypothalamic leptin−melanocortin pathway. *Indicate genetic obesity syndromes

We and others have demonstrated that human obesity can result from a multiplicity of defects in the leptin–melanocortin pathway (Fig. 1). Generally these disorders are rare, being found in 1–5 % of patients with severe obesity (own observations). Individuals with homozygous loss-of-function mutations in the genes encoding leptin and the leptin receptor have a normal birthweight but exhibit rapid weight gain in the first few months of life resulting in severe obesity (Montague et al. 1997; Farooqi et al. 2002). Characteristic features are an intense drive to eat (hyperphagia) as well as impaired satiety with food-seeking behavior soon after the end of a meal. Recombinant human leptin injections are effective in these individuals leading to normalization of hyperphagia, enhanced satiety, and weight loss (Farooqi et al. 1999). Leptin is also involved in mediating food reward. In rodents, leptin receptors are expressed in the mesolimbic brain regions which mediate the rewarding properties of food and drugs (Barge-Schaapveld et al. 2011). Leptin administration decreases the firing threshold of dopaminergic neurons within this system, while long-term RNAi-mediated knockdown of Lepr in the ventral tegmental area has been shown to increase food intake and sensitivity to highly palatable food (Hommel et al. 2006). In leptin-deficient humans, images of food (compared to non-food images) are associated with a marked increase in neuronal activation in the ventral striatum imaged using functional MRI. This response was normalized by 7 days of leptin treatment (Farooqi et al. 2007a) before significant weight loss consistent with the view that activation in the ventral striatum does not directly encode the “liking” but rather the motivational salience, or “wanting,” of food.

In leptin-deficient children and adults, basal metabolic rate, total energy expenditure, and free-living energy expenditure are appropriate for body composition (Farooqi et al. 2007c). Ravussin and colleagues showed that before weight loss, leptin-deficient adults and matched controls had similar energy expenditures. However, after weight loss, controls had energy expenditures lower than expected for their new weight and body composition (Galgani et al. 2010). This is a well-known phenomenon, often called the metabolic adaptation to weight loss, i.e., a decrease in metabolic rate beyond that expected on the basis of the decrease in fat-free mass and fat mass. Therefore, the absence of this change in energy expenditure in some leptin-deficient adults is consistent with an effect of leptin on energy expenditure.

Body composition measurements show that leptin deficiency is characterized by the preferential deposition of fat mass (compared with lean mass), and weight loss leads to a preferential loss of fat mass. In rodents, leptin stimulates fatty acid oxidation in skeletal muscle via the stimulation of AMP kinase activity (Minokoshi et al. 2002). In leptin-deficient humans, impaired fat oxidation has been demonstrated by chamber calorimetry (Galgani et al. 2010). Ozata et al. (1999) have reported abnormalities of sympathetic nerve function in leptin-deficient adults consistent with defects in the efferent sympathetic limb of thermogenesis. Evidence from rodents and humans suggests that leptin is necessary for the normal biosynthesis and secretion of thyrotropin-releasing hormone and that complete leptin deficiency is associated with a moderate degree of hypothalamic hypothyroidism characterized by low free thyroxine and high serum thyroid-stimulating hormone, which is bio-inactive (Gibson et al. 2004).

## Insights from genetics—melanocortinergic circuits

Leptin signaling modulates energy balance through a combination of melanocortin-dependent and -independent pathways. In humans, null mutations in POMC lead to hyperphagia, early-onset obesity, isolated adrenocorticotropin (ACTH) deficiency, and hypopigmentation of skin and hair (Coll et al. 2007). Heterozygous null mutations in *POMC* and loss-of-function mutations in α- and β-melanocyte-stimulating hormone (α- and β-MSH) significantly increase obesity risk but are not invariably associated with obesity (Coll et al. 2004).

Prohormone convertase 1 (PCSK1) is an enzyme involved in the cleavage of POMC into ACTH, which is then further cleaved to make α-MSH by carboxypeptidase E. Humans lacking PCSK1 are severely obese and have glucocorticoid deficiency, hypogonadotropic hypogonadism, and postprandial hypoglycemia, which occurs as a result of impaired processing of proinsulin to insulin by PCSK1 (Farooqi et al. 2007b). Elevated plasma levels of proinsulin and 32–33 split proinsulin in the context of low levels of mature insulin provide the basis for a diagnostic test for this disorder (Jackson et al. 1997).

We and others have reported that mutations in MC4R are found in 5–6 % of patients with severe early-onset obesity (Farooqi et al. 2003; Stutzmann et al. 2008) and at a frequency of approximately 1/1,000 in the general UK population, making this one of the most common human genetic diseases. Functionally significant MC4R mutations are inherited in a co-dominant manner, with variable penetrance and expression in heterozygous carriers. Most naturally occurring disease-causing MC4R mutations disrupt normal expression and trafficking of the receptor to the cell surface (Vaisse et al. 2000). The mechanism of GPCR dysfunction has potential interest, as we have shown that pharmacological chaperones can increase the cell surface expression and signaling of mutant GPCRs. We have previously characterized human MC4R deficiency and reported hyperphagia, increased lean mass, and increased linear growth and demonstrated a genotype−phenotype correlation with the degree of receptor dysfunction in vitro predicting all aspects of the phenotype, including ad libitum energy intake (Farooqi et al. 2007c).

We have shown that both increase and decrease in central melanocortin signaling influence blood pressure in humans and that the effects are not explained by changes in circulating insulin levels or insulin sensitivity (Greenfield et al., 2009). MC4R-deficient patients with long-standing decreases in melanocortinergic tone have a lower prevalence of hypertension and lower systolic and diastolic blood pressures. These changes are associated with reduced sympathetic nervous system activity. Also, administration of a melanocortin receptor agonist in obese volunteers increases blood pressure. Thus, central melanocortin signaling appears to play an important role in the regulation of blood pressure and its coupling to changes in weight. With weight gain, leptin levels increase, thereby increasing signaling through MC4R. As MC4R neurons synapse with preganglionic sympathetic neurons which regulate vascular tone, increased signaling through MC4R can lead to an increase in blood pressure. This mechanism explains why reduced melanocortin signaling in people with loss-of-function MC4R mutations, may be associated with reduced blood pressure.

## Insights from genetics—molecules downstream of MC4R and other mechanisms

Several lines of evidence suggest that Brain-derived neurotrophic factor (BDNF), a nerve growth factor which activates signaling by the tyrosine kinase receptor tropomycin-related kinase B (TrkB), and SIM1 may lie downstream of MC4R signaling in the paraventricular nucleus. Haploinsufficient mice and mice in which BDNF has been deleted postnatally are obese with hyperphagia and hyperactivity; this unusual combination of phenotypes is also seen in individuals with genetic disruption of BDNF or its receptor TrkB (Gray et al. 2006; Yeo et al. 2004).

Single minded 1 (SIM1) is a transcription factor involved in the development of the paraventricular and supraoptic nuclei of the hypothalamus. A de novo balanced translocation between chromosomes 1p22.1 and 6q16.2, which disrupts SIM1 (Holder et al. 2000), and missense mutations in SIM1 cause severe obesity and a variable phenotype of developmental delay (Ramachandrappa et al. 2013). The transcriptional targets of SIM1 are unknown, but one potential target is the neuropeptide oxytocin. Oxytocin mRNA levels are reduced in line with *Sim1* gene dosage in mouse models of Sim1 deficiency. The hyperphagia of *Sim1*-haploinsufficient animals is ameliorated by oxytocin administration and accentuated by the administration of oxytocin receptor antagonists (Kublaoui et al. 2008).

Recent studies have suggested a connection between ciliary function and leptin signaling (Seo et al. 2009). Conditional postnatal knockout of proteins involved in intraflagellar transport in mice results in hyperphagia and obesity. This phenotype is recapitulated when the loss of cilia is limited to neurons and when it is specifically targeted to POMC neurons. As several human obesity disorders disrupt genes involved in ciliary function (e.g., Alström syndrome and Bardet−Beidl syndrome) (Tobin and Beales 2007), the role of cilia in key neuronal populations involved in energy homeostasis is likely to be the subject of much future research.

## Finding the missing heritability in human obesity

Next-generation sequencing technologies are currently being used to undertake CNV analysis (Walters et al. 2010) and whole exome sequencing in obese cohorts and populations (Gill et al. 2014). The aim of these studies is to find low-frequency/rare genetic variants, but assessing the potential pathogenicity of those variants remains a major challenge. While statistical and computational methods are continually emerging to address these challenges, as with other hypothesis-free genetic approaches the technique is ultimately only as powerful as the genetic material being interrogated. Initial methodological approaches have tended toward sequencing related individuals, or sequencing individuals at opposing extremes of the phenotypic spectrum. The contribution of epigenetic modulation and of non-coding genetic variation to the missing heritability of obesity remains to be explored.
